# Analgesic Action of Catechin on Chronic Constriction Injury–Induced Neuropathic Pain in Sprague–Dawley Rats

**DOI:** 10.3389/fphar.2022.895079

**Published:** 2022-08-11

**Authors:** Ahmed I. Foudah, Mohammed H. Alqarni, Sushma Devi, Akanksha Singh, Aftab Alam, Pravej Alam, Sima Singh

**Affiliations:** ^1^ College of Pharmacy, Prince Sattam Bin Abdulaziz University, Al-Kharj, Saudi Arabia; ^2^ Guru Nanak Institute, Hema Majra Road, Mullana, Ambala, India; ^3^ Prin, K. M. Kundnani College of Pharmacy, Rambhau Salgaonkar Marg, Cuffe Parade, Mumbai, India; ^4^ Department of Biology, College of Science and Humanities, Prince Sattam Bin Abdulaziz University, Al-Kharj, Saudi Arabia; ^5^ IES Institute of Pharmacy, IES University Campus, Kalkheda, Ratibad Main Road, Bhopal, India

**Keywords:** neuropathic pain, catechin, herbal, inflammation and pain, chronic constriction injury

## Abstract

Chronic neuropathy is a common and debilitating problem that poses a significant challenge to health care worldwide. Natural compounds have received considerable attention as potential sources of new drugs for the treatment of neuropsychiatric pain. Catechin is a well-known novel flavonoid with several therapeutic properties, notably in neurodegenerative diseases. The current study is designed to investigate the role of catechin in neuroprotective activity in the chronic constriction injury (CCI) model. Apparently, healthy adult male Sprague–Dawley rats weighing 160–190 g (8 weeks old) were selected and grouped into the following: sham (distilled water), CCI group (CCI), standard [CCI + pregabalin (10 mg/kg, p.o.)], and test catechin [CCI + catechin (50 and 100 μg/kg p.o.)] for 28 days. Behavioral, thermal, and mechanical changes were evaluated. The results showed that mechanical allodynia and thermal hyperalgesia were reduced in the catechin-treated group when compared with the CCI group. In addition, the relationship between the analgesic effect of catechin and the expressions of TNF-α, IL-6, and IL-β was established. The results showed that catechin reversed the signs of neuropathic pain. It also decreased the levels of TNF-α, IL-6, and IL-β in the rat brain. Therefore, the results suggested that catechin has promising potential in the treatment and management of neuropathic pain by decreasing the levels of NF-κβ–regulated inflammatory cytokines in the chronic constriction injury model.

## Introduction

Neuropathic pain is a chronic and severe condition produced by the somatosensory nervous system (Singh et al., 2017). It includes the central nervous system (CNS) and the peripheral nervous system (PNS). CNS or PNS injuries result in unpleasant somatosensory experiences (Bernetti et al., 2021). Some types of peripheral nerve damage cause long-lasting localized neuropathic pain. These injuries can be caused by trauma, infections, inflammation, tumors, metabolic diseases, or endocrine diseases (Baron, 2006). Researchers estimate that neurological pain affects between 3 and 17% of the world population (Van Hecke et al., 2014). It causes a substantial burden on individuals, societies, and economics. It affects about 10% of the population in the United States and can last for years or even a lifetime after an initial injury (Corso et al., 2006).

The pathophysiological mechanism underlying neuropathic pain is reported to be highly complicated [5]. Neuropathic pain is caused by neuro-inflammatory processes that affect nerve tissue and regulate the inflammatory response. The relationship between neuropathic pain and peripheral nerve injury is based on the TNF-α–driven pro-inflammatory cytokine mechanism (Myers et al., 2006). Other pro-inflammatory cytokines are also involved, such as interleukin (IL)-6 and IL-1β. The secretion of conventional mediators of inflammation such as prostaglandins and iNOS. The upregulation of these factors in the nervous system leads to appalling results (Myers et al., 2006).

The activation of glial cells leads to the release of tumor necrosis factor-alpha (TNF-α) and IL-1β. Nuclear factor κB (NF-κB) transcriptionally regulates the release of cytokines during the inflammatory process (Fu et al., 2010). Following nerve injury, the activity and expression of NF-κB have been observed in the dorsal root ganglia and sciatic nerve (Ledeboer et al., 2005). The role of NF-κB is quite vital. Although several other factors such as IL-6 and IL-1β also seem to contribute to nerve root injury in sciatica. TNF-α alone has been reported to cause neuropathic changes and mechanical allodynia, mimicking the primary aspect of neuropathic conditions in humans (Takahashi et al., 2006).

Currently, neuropathic pain treatments are only restricted to surgical intervention and pharmacological approaches, limiting their effectiveness (Carvajal et al., 2018). The available approved therapies focus mainly on two well-known analgesics: opiates and nonsteroidal anti-inflammatory drugs (NSAIDs) (Moore et al., 2015). These therapies have fewer pharmacological effects and more side effects. The underlying intricacy of pain transmission and processing makes it extremely difficult to discover new targets and analgesic medications. The lack of suitable therapies has encouraged our research to identify novel therapies for neuropathic pain. Alternative treatments that offer more pharmacological effects and lesser side effects are needed to manage pain.

Traditional herbal medicine has been shown to have powerful antinociceptive effects on chronic pain with lesser adverse effects than existing treatment therapies. Catechin is a group of flavonoid compounds derived from *Camellia sinensis* var. sinensis plant (Unachukwu et al., 2010). Increasing evidence have revealed that catechin has several protective properties against the treatment of fever, inflammatory disorders, wounds, and cancers in different parts of the world. It is well known for its anti-inflammatory capacity. It can inhibit the expression of pro-inflammatory cytokines (Vazquez Prieto et al., 2015;Noll et al., 2013) by inhibiting nuclear factor-kappa B (NF-κB) (Suhail et al., 2019). These observations strongly suggest that catechin can be effective in the treatment of neuropathic pain in rats by determining its potential to inhibit the expression of various pro-inflammatory cytokines like IL-1β, IL-6, and TNF-α. However, this antinociceptive efficacy of catechin on a chronic constriction injury (CCI)–induced model remains to be clarified.

## Materials and Methods

### Drugs and Chemicals

Catechin was obtained as a gift sample from Ayurvet Ltd., Baddi, India. The ELISA kit was purchased from Cayman Chemical (Michigan, United States). Pregabalin (Mylan Labs, India), ketamine (Themis Pharmaceuticals Ltd., India), and xylazine (Med Vet, Mumbai, India) were purchased from commercial sources. All chemicals were of analytical grade.

### Experimental Animals

Apparently, healthy adult male Sprague–Dawley rats (8 weeks old) weighing 160–190 g were obtained from the Animal Facility of Swift School of Pharmacy, Rajpura, Punjab, India. After the Institutional Animal Ethics Committee (IAEC) approval, studies were carried out, with approval no.-1616/PO/Re/S/12/CPCSEA (protocol no. IAEC/19/005). All experiments were carried out according to the current CPCSEA guidelines, New Delhi. The selected animals were housed and kept in a climate-controlled environment with a constant temperature of 25 ± 2°C, relative humidity of 55%, a 12:12 h light:dark cycle and had free access to food and water, *ad libitum*. In this study, different doses were administered according to the preliminary investigations as shown in Table 1.

**TABLE 1:** Grouping of animals.

Groups	Subjects	Treatment given
Group I	Sham	Vehicle (distilled water)
Group II	CCI groups	CCI
Group III	Standard	CCI + pregabalin (10 mg/kg, p.o.)
Group IV	Test catechin	CCI + catechin (50 μg/kg p.o.)
Group V	Test catechin	CCI + catechin (100 μg/kg p.o.)

#### Chronic Constriction Injury Model

The sciatic nerve developed peripheral mononeuropathy in rats according to the method previously reported by Mao et al. (1993) and Shahid et al. (2017). Rats in both the ligation and sham groups (n = 6/group) were anesthetized with a ketamine:xylazine mixture in a ratio of 4:1. A blunt dissection revealed the common sciatic nerve through the biceps femoris in the center of the thigh. Approximately 7 mm of the sciatic nerve was removed from the adherent tissue proximal to the trifurcation. Four ligatures (4-0 chromic gut) were loosely wrapped around it with an interval of about 1 mm. The nerve was 4–5 mm long when it was impacted. The sham group had a similar dissection, except that the sciatic nerve was not ligated. All surgical procedures were performed under standard sterile conditions. Primary care was taken for the wound by applying povidone iodine ointment. The animals were inspected every 2 days during the first 14 days and then weekly for 28 days. During these examinations, each rat was placed on a table and carefully studied for a minute or two for changes in gait, the posture of the affected hind paw, the skin condition of the affected hind paw, and the amount of autonomy if present.

#### Behavioral Methods to Measure Pain-Like Behaviors

The nociceptive stimulus is the most commonly used method to quantify nociception in animal studies. Behavioral tests have been performed in CCI-induced animals after a previously reported investigation before pain induction, on the 0th, 3rd, 7th, 14th, 21st, and 28th day after surgery. The animals were sacrificed on the 28th day. The sciatic nerve, brain, and spinal cord of the lumbar region were isolated with snap freeze using liquid nitrogen and stored at −80°C. The total value was also estimated for biochemical, molecular, and histological parameters.

#### Test for Spontaneous Pain Using a Neutral Plate

To estimate the level of spontaneous pain (ongoing pain without apparent external stimuli), the neutral plate method was used by following the method published by Yoon et al. (1999) and Lee et al. (2000). Each animal was placed on an aluminum plate maintained at an ambient temperature (25 ± 0.5°C) beneath an inverted, clear plastic cage for 5 min. The duration of lifting was recorded for the next 5 min. The reaction time for the animal to lick the paw or the withdrawal of the paw not related to the general movement was taken as the latency period.

#### Test for Cold Allodynia Using a Cold Plate

For the measurement of cold allodynia using a cold plate, each rat was placed on a brass plate kept at a cold temperature (5 ± 1°C) (Robinson and Meert, 2005). It was allowed to acclimate for 5 min. After 5 min of adaptation, the cumulative duration of time that the rat held its foot off the floor for the next 5 min was recorded (Ruan et al., 2018).

#### Test for Mechanical Allodynia Using the von Frey Test

Automated von Frey filaments are fine gauge metal wires used to test the rat’s sensitivity to mechanical stimuli by following the previously reported method (Li et al., 2019; Nakamura et al., 2021). Rats were kept in acrylic cages with a wire mesh grid on top. A probe was placed on the hind paw for testing, avoiding less sensitive footpads. Stimulation was maintained until the paw was removed or gradually increased. In this technique, a maximum acceptable force can be calculated. The procedure was performed three times on each paw, with a minimum of a 10-s gap between each test. The final reading was taken as the average of three measurements.

#### Locomotor Activity Using an Actophotometer

Locomotor activity was measured using an actophotometer, as reported by Nishat Fathima et al. (2020). An actophotometer provided with a digital counter with infrared sensors and a computerized counter and light source was used to measure locomotor activity (horizontal movement) of animals. Each animal was placed in the actophotometer for 5 min, and the basal activity score was recorded for all animals. Total counts/5 min per animal were used to measure the locomotor activity.

#### Evaluation of the Antinociceptive Potential of Catechin

Apparently, healthy adult male Sprague–Dawley rats (n = 6) were anesthetized with a 4:1 combination of ketamine and xylazine (1 ml/kg, i.p. supplemented). The common sciatic nerve was revealed by blunt dissection through the biceps femoris in the center of the thigh. Approximately 7 mm of the sciatic nerve was removed from the adherent tissue proximal to the trifurcation. Four ligatures (4-0 chromic gut) were loosely wrapped around it with an interval of about 1 mm. The nerve was 4–5 mm long when it was impacted. Under the magnification of ×40, it was observed that ligatures were tied with extreme care. The required degree of constriction slowed but did not stop the blood flow through the superficial epineurial veins and occasionally caused a slight transient spasm in the muscle around the exposure. The incision was stitched up in layers. The sham group had a similar dissection, except that the sciatic nerve was not ligated. The animals were examined and treated with catechin from day 14 to day 28.

### Biochemical Parameters

#### Estimation of the Transcription Factor Nuclear Factor κB by ELISA

Transcription factor analysis was performed with an ELISA kit (Cayman Chemical, Michigan, United States) that allowed the detection of NF-κB (Costa et al., 2008). The bottom of the well was immobilized with a particular double-stranded DNA (dsDNA) sequence, including an NF-κB response element. The nuclear extract contained NF-κB, which binds to the NF-κB response element. Subsequently, it was detected by adding a specific antibody directed against NF-κB. The brain, spinal cord, and sciatic nerve samples were homogenized in 100 µl of ice-cold hypotonic lysis buffer per milligram of tissue. Five hundred microliters of hypotonic buffer was supplied with 25 µl of Nonidet after centrifugation for 10 min. The pellets were mixed with P-40, and the combination was centrifuged at 14,000 rpm for 2 min at 4°C. The pellets were then suspended in 50 µl of hypertonic lysis buffer. They were incubated at 4°C for 30 min with shaking. The supernatant containing the nuclear extracts was kept at −80°C after centrifugation at 14,000 rpm for 10 min at 4°C. On the ELISA plate, 10 µg of nuclear protein extract was added. Then, it was incubated at room temperature for 1 h. A primary antibody recognizes an epitope in p65. After 1 h, the HRP substrate was added. The reaction was stopped after 10 min. The absorbance was recorded on a microplate reader at 450 nm. A cell lysate containing the transcription factor NF-κB (human p65) was used as a positive control.

#### RNA Extraction and Reverse Transcription–Polymerase Chain Reaction for the Detection of Messenger RNA for IL-1 β, IL-6, and TNF-α

*RNA isolation*. The mRNA levels were analyzed using the reverse transcription (RT-PCR) approach by following a previously described method (Konturek et al., 2000). The brain was diced and chopped. The samples were disrupted in liquid nitrogen using a mortar and pestle. The guanidium isothiocyanate–phenol–chloroform TRIzol technique extracted the total cytoplasmic RNA from tissue samples. The RNA was washed with 70% ethanol after being precipitated with isopropyl alcohol. It was then treated for 45 min with an RNase inhibitor. After resuspending the RNA at 65°C for 15 min, a Qiagen RNA isolation kit was used to purify the RNA further. The mixture was then processed with RNase-free DNase according to the manufacturer’s instructions. The RNA was resuspended in RNase-free water after precipitation. The absorbance at the 260-nm wavelength was used to determine the concentration. The RNA samples were kept at −80°C until analyzed.

*cDNA preparation*. Reverse transcriptase (Cat. RR014A, Takara Bio. Inc., CA, United States) and oligo-(dT) primers were used to make single-stranded cDNA from 5 µg of total cellular RNA, according to the instructions given by the manufacturer. Of the total RNA, 5 µg was heated at 65°C for 5 min to uncoil. A reaction combination of 50 U Moloney murine leukemia virus reverse transcriptase (M-MLV RT), 0.3 µg oligo (dT) primer with 1 µl RNase block ribonuclease inhibitor (40 U/µl), and 2 µl of 100 mM dATP, dTTP, dGTP, and dCTP master mix with 5 µl 10× buffer was used to make the cDNA. The cDNA (2 µl) was amplified in a 50-µl reaction volume containing 2 U Taq polymerase, 200 µM dNTP, 1.5 mM MgCl_2_, 5 µl 10× polymerase chain reaction buffer, and specific primers used at a final concentration of 0.5 µM. A DNA thermal cycler (Veriti^®^ 96-well, Applied Biosystems, CA, United States) was used to amplify the polymerase chain reaction mixture. Band intensities were compared against those expressed constitutively (Konturek et al., 2003; Brzozowski et al., 2005).

#### Electrophysiology of Nerves

The motor nerve conduction velocity was measured as an index of the speed of conduction of an electrical impulse through a nerve using a Student Physiograph by following a previously described method (Kumar et al., 2007). The sciatic and tibial nerves were stimulated with supramaximal stimulation, 0.5 V, single stimulus, and square wave pulses for a duration of 0.2 ms using bipolar needle electrodes (26 × 1/2 gauge). The paired steel needle electrodes were placed percutaneously at 1 cm between the reference and the stimulation or recording electrodes. Then, the stimulating electrodes were implanted in the sciatic notch and knee. On the tiny muscles of the hind paw dorsum, recording electrodes were placed. Surface electrodes attached to a potential BioCoupler were implanted in the paw to detect the motor response. A clip was used to secure a ground electrode to the calf muscle. The body temperature was maintained at 37°C throughout the experiment. The Student Physiograph software was used for recordings caused by motor fiber activation (LabChart 7.3.7). In milliseconds, the latency (the time between stimulation and the beginning of the reaction) was assessed. The distance between the two stimulating electrodes was measured in micrometers and converted to meters. The following formula was used to calculate the conduction velocity of the motor neuron.

$$Motor\;nerve\;conduction\;velocity\;\left(m/s\right)\;=\frac{\left(Dis\tan ce\;between\;the\;nerve\;stimulation\;po\mathrm{int}\right)\;in\;meters}{\left(Sciatic\;M-wave\;latency-tibial\;M\;wave\;latency\right)\;in\;\sec onds}$$

### Statistical Analysis

All statistical tests were performed using the GraphPad Prism software, version 8 (GraphPad Software Inc., La Jolla, CA, United States). The data were treated with appropriate analysis of variance (ANOVA). All values were expressed as mean ± SEM. *p* < 0.001 was considered statistically significant.

## Results

### General Observations

After surgery, no signs of autotomy or loss were observed. Following nerve ligation, no motor impairment was observed. The sham animals acted in the same way on both the ipsilateral and the contralateral sides. Throughout the testing period, no significant differences were found in either modality.

### Assessment of Spontaneous Pain Using a Neutral Plate

**FIGURE 1:**
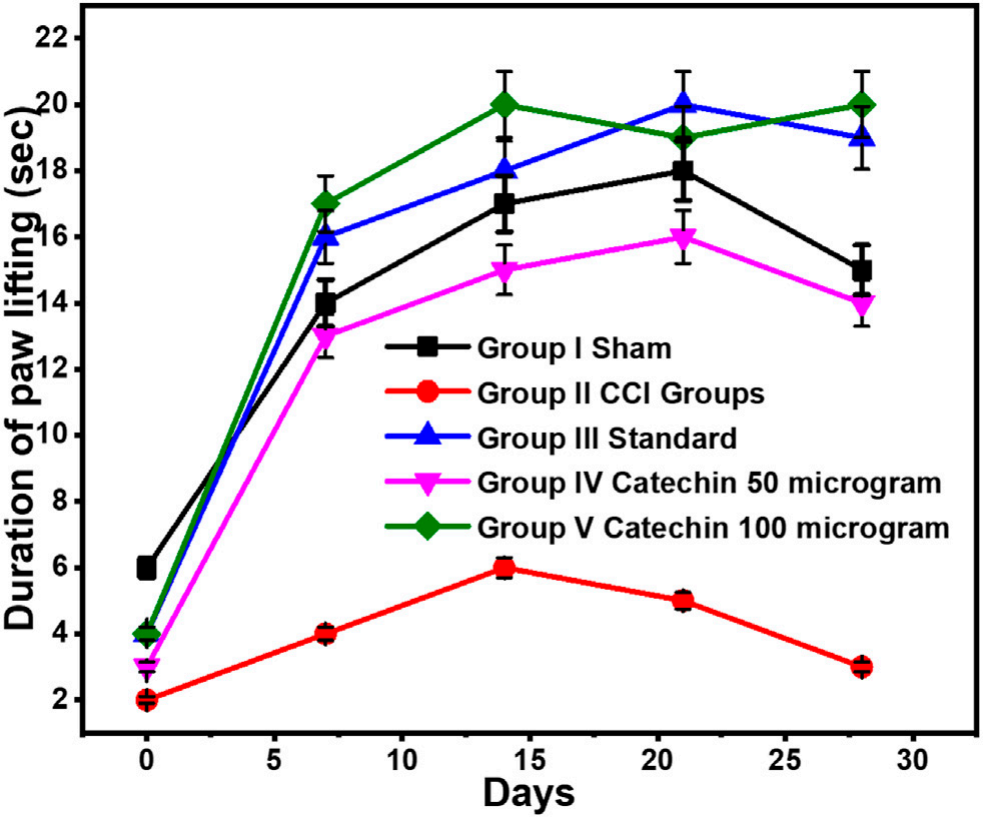
Assessment of spontaneous pain using a neutral plate. Values are expressed as mean ± SEM for six animals in each group. Significance was determined by ANOVA. *p* < 0.001 when compared with sham and CCI.

CCI resulted in the significant development of spontaneous pain, as indicated by the increase in the duration of paw lifting on a neutral plate. On day 0, the course of lifting the paw in the sham group was 6 ± 0.3 s and 14 ± 0.07 s after 1 week. Throughout the experiment, a significant difference in paw lifting was observed between the sham group and the CCI group. Pregabalin (10 mg/kg) did not attenuate the CCI-induced increase in the duration of paw lifting. The time of paw lifting improved significantly in the groups receiving catechin 50 and 100 μg/kg i.p. in a dose-dependent manner. With catechin 50 μg/kg i.p., the duration of paw lifting was found to be 16 ± 0.8 s and 14 ± 0.7 s on the 21st day and the 28th day, respectively. These values were reduced to 19 ± 0.95 s and 20 ± 1 s on the 21st and 28th days, respectively, with catechin 100 μg/kg, as shown in Figure 1.

### Assessment of Cold Allodynia Using Cold Plate Technique

Each animal was placed on an aluminum plate cooled to 4 ± 2°C with ice beneath an inverted clear plastic cage and was allowed to acclimate for 5 minutes. It was found to be significantly increased in the CCI group when compared with the sham group. On day 7, the duration of paw lifting in the sham group was 15 ± 0.75 s. This was significantly more than the duration of paw lifting in the positive control group, which was found to be 3 ± 4.03 s. However, pregabalin treatment slowed this increase in the CCI group when lifting their paws dose-dependently. On the 21st day, the duration of paw lifting was found to be 4 ± 14.01 s in the CCI group. This was further increased to 22 ± 7.47 s in the group receiving pregabalin (10 mg/kg p.o.) on day 21. On the 28th day, it was found that the duration of paw lifting was 6 ± 13.61 s in the CCI group, which had increased to 23 ± 7.02 s in the group that received pregabalin 10 mg/kg p.o.

**FIGURE 2:**
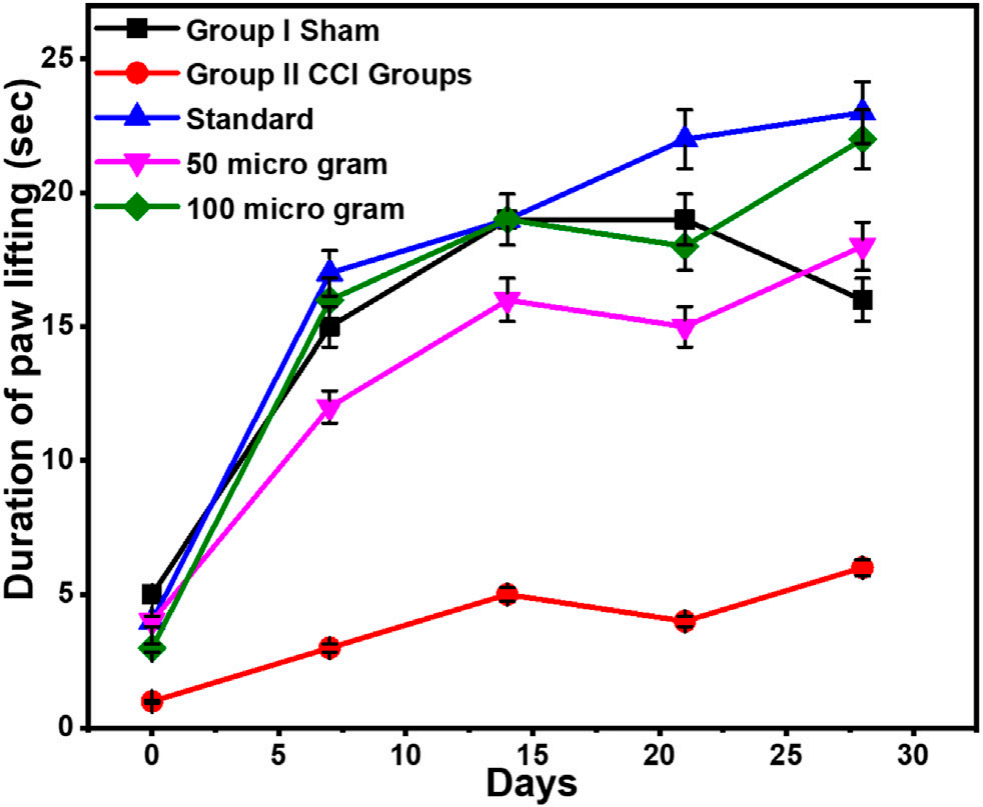
Assessment of cold allodynia using the cold plate technique. Values are expressed as mean ± SEM for six animals in each group. Significance was determined by a two-way analysis of variance. *p* < 0.001 when compared with sham and CCI.

This difference had been consistent throughout the experiment. However, catechin treatment with 50 μg/kg i.p. reversed this increase in the duration of paw lifting. It was found to be 15 ± 3.12 s and 18 ± 3.05 s on the 21st and 28th days, respectively. On the 21st and 28th days, catechin 100 μg/kg i.p. reduced the duration of paw lifting to 18 ± 3.56 s and 22 ± 4.15 s, respectively, in the treatment group, as shown in Figure 2.

### Assessment of Mechanical Allodynia Using the von Frey Test

The sciatic nerve resulted in a significant decrease in the threshold for paw withdrawal in the von Frey test. This indicates the development of mechanical allodynia. On day 7, the CCI group reduced the paw withdrawal threshold to 3 ± 12.13 g, which lasted throughout the experiment. This decrease was significantly low compared with the paw withdrawal threshold. It was 18 ± 12.86 g in the treatment group that received pregabalin 10 mg/kg of p.o. On the 28th day, the withdrawal threshold was found to be 4 ± 8.19 g in the CCI group. In addition, it was increased to 20 ± 12.86 g in the treatment group that received pregabalin 10 mg/kg p.o. on day 28.

**FIGURE 3:**
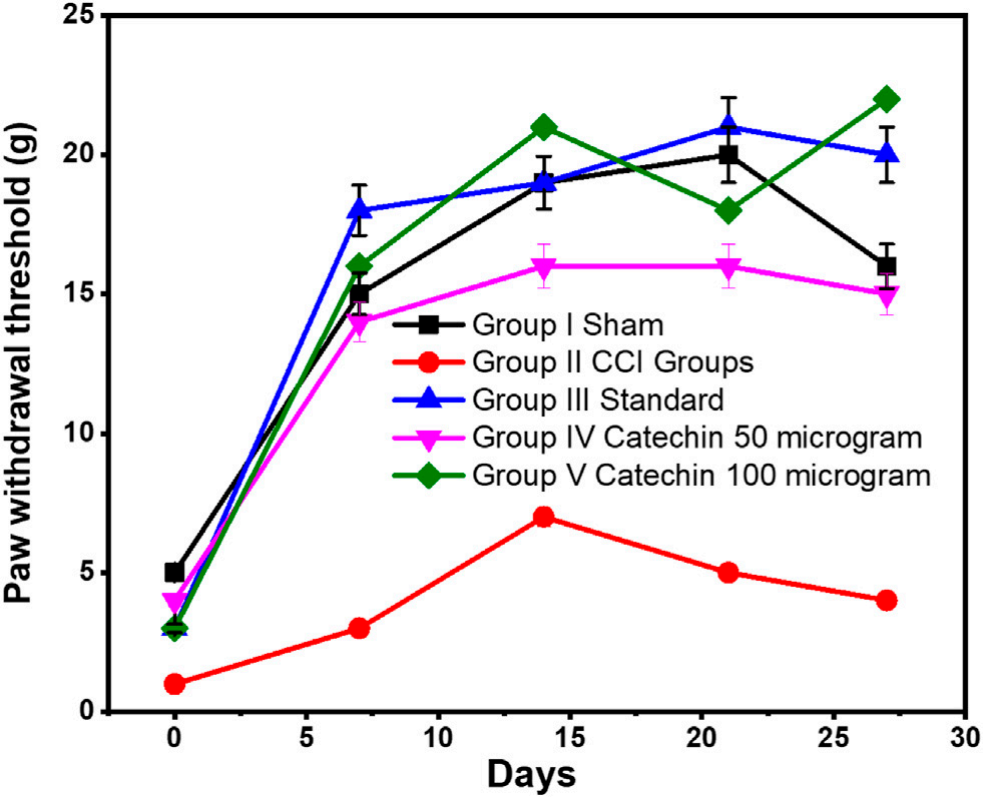
Assessment of mechanical allodynia using the von Frey test. Values are expressed as mean ± SEM for six animals in each group. Significance was determined by two-way analysis of variance. *p* < 0.001 when compared with sham and CCI.

The paw withdrawal threshold improved to 15 ± 6.47 g in the treatment group receiving catechin 50 μg/kg i. p. on the 28th day. This increased even more to 22 ± 24.15 g on the day the group received 100 μg/kg catechin, as shown in Figure 3.

### Assessment of Locomotor Activity Using an Actophotometer

In the present study, CCI-induced behavioral alteration started on the 3rd day and lasted throughout the experiment. Catechin had facilitated the increase in the sensation of nociceptive pain, electrophysiological changes, and biochemical changes. The antinociceptive effect of the selected catechin doses was indicated by their potential to attenuate spontaneous pain (neural plate). The duration of paw lifting, when placed on a neutral plate, was found to decrease in the treatment group when compared with the CCI group in a dose-dependent manner on days 21 and 28. Catechin also attenuated cold allodynia, as indicated by the cold plate. The duration of paw lifting decreased in a dose-dependent manner in the last 2 weeks. Catechin was found to significantly increase the paw withdrawal threshold on days 21 and 28 when compared with sham. Treatment with CCI and catechin did not significantly reduce locomotor activity/5 min on any of the assessment days when compared with sham and CCI, respectively (Table 2).

**TABLE 2:** Effect of catechin on locomotor activity using an actophotometer.

Time interval (in days)	Ambulation counts/5 min			
	Sham	CCI	Catechin 50 μg/kg	Catechin 100 μg/kg
**Pre**	337.17 ± 16.858	257.43 ± 12.871	362.24 ± 18.112	360.04 ± 18.002
**7th**	378.31 ± 18.915	350.82 ± 17.541	391.31 ± 19.56	312.00 ± 15.602
**14th**	336.23 ± 16.811	285.13 ± 14.256	362.13 ± 18.1065	299.33 ± 14.966
**21st**	325.17 ± 16.258	266.82 ± 13.341	316.00 ± 15.8	239.26 ± 11.963
**28th**	330.27 ± 16.513	241.83 ± 12.09	307.44 ± 15.372	222.47 ± 11.123

Values are expressed as mean ± SEM, for six animals in each group. Significance was determined by two-way analysis of variance. #: *p* < 0.001 when compared with sham and CCI.

### Effect of Catechin on Nuclear Factor κB Activation

**FIGURE 4:**
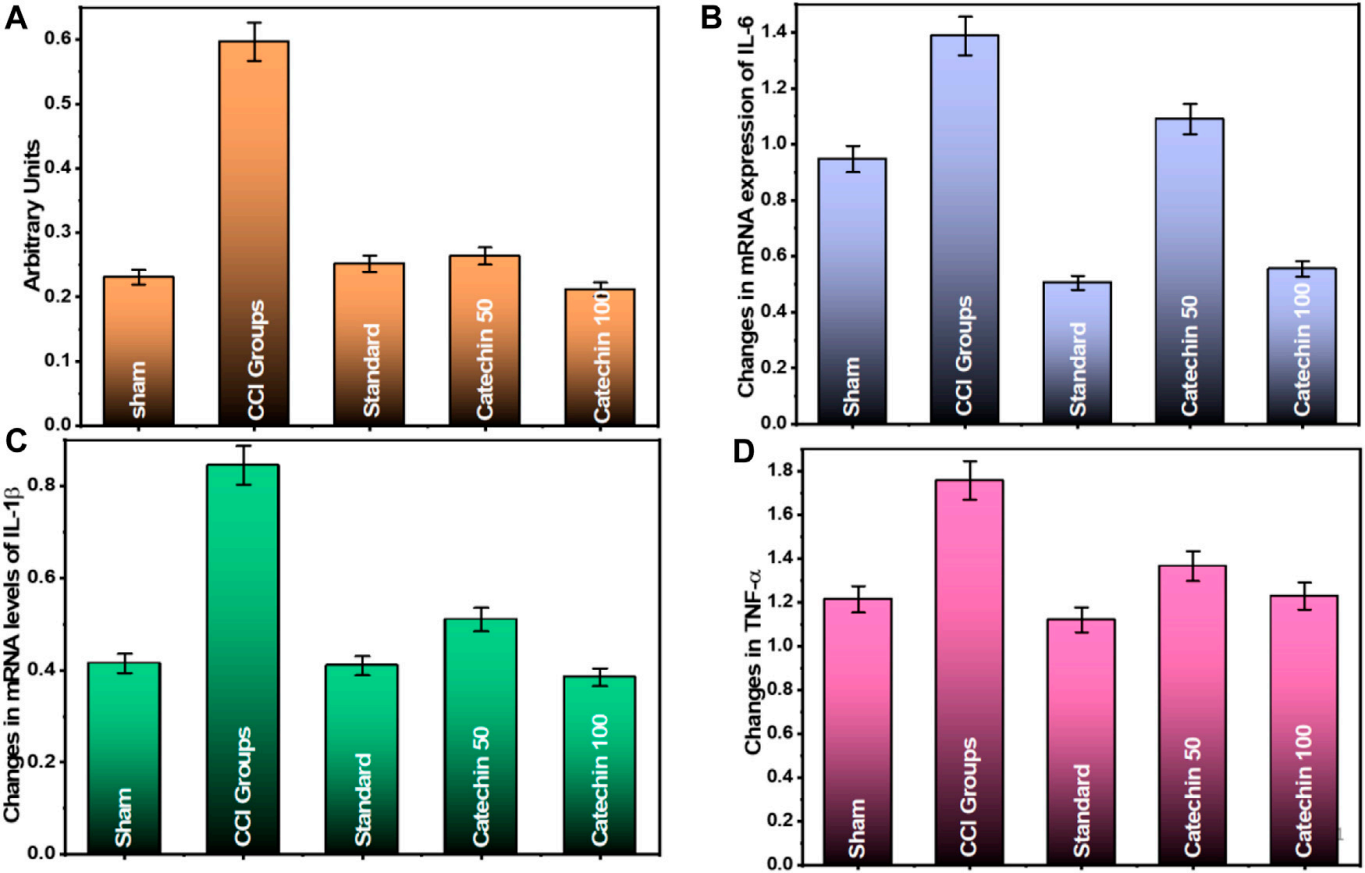
Effect of catechin on **(A)** NF-κB activation; **(B)** mRNA expression of IL-6; **(C)** mRNA expression of IL-1β; and **(D)** mRNA expression of TNF-α.

The activated NF-κB test demonstrated that the DNA binding activity of NF-κB component p65 was elevated in the sciatic nerve of CCI rats. According to the results on the 28th day after the injury, in the CCI group, NF-κB activation was found to be 0.597 ± 0.020 (arbitrary unit). On the contrary, in the control group, it was found to be 0.231 ± 0.679 (arbitrary unit). An increase in the NF-κB DNA binding activity was significantly reduced by administering catechin (50 and 100 μg/kg) to rats. An average of 0.264 ± 0.035 (arbitrary unit) and 0.212 ± 0.073 (arbitrary unit) was reported in the treatment groups that received 50 and 100 μg/kg catechin, respectively, as shown in Figure 4A. CCI-induced neuropathic pain has been shown to increase NF-kB levels. This increase in NF-kB activation is believed to be associated with an improved nociceptive response. It was evidenced by the increase in inflammatory cytokines production that causes inflammation and pain. Compared with the CCI group, catechin administration for 14 days resulted in a dose-dependent reduction in NF-κβ in the sciatic nerve.

### Effect of Catechin on mRNA Expression of IL-6, IL-1β, and TNF-α

The level of IL-6 mRNA was found to be 1.387 ± 0.057 in the CCI group and 0.947 ± 0.055 in the sham group. These signals decreased significantly to 1.09 ± 0.081 and 0.554 ± 0.071 in a dose-dependent manner when treated with catechin at 50 and 100 μg/kg, respectively (Figure 4B).

The IL-1 mRNA levels of IL-1β were found to be 0.845 ± 0.012 in the CCI group and 0.415 ± 0.012 in the sham group. These signals decreased significantly to 0.510 ± 0.065 and 0.383 ± 0.074 in a dose-dependent manner when treated with catechin at 50 and 100 μg/kg, respectively (Figure 4C).

The mRNA level of TNF-α was found to be 1.756 ± 0.121 in the CCI group and 1.213 ± 0.065 in the sham group. These levels decreased significantly to 1.364 ± 0.054 and 1.228 ± 0.077 in a dose-dependent manner in the treatment groups that received catechin 50 and 100 μg/kg, respectively (Figure 4D).

### Effect of Catechin on Motor Nerve Conduction Velocity

On the penultimate day before sacrifice, chronic constriction damage resulted in a considerable loss of neuronal electrical activity, as demonstrated by a decrease in the motor nerve conduction velocity (MNCV) (56.24 ± 1.12) when compared with sham (75.66 ± 1.65). Administration of catechin (50 and 100 μg/kg i.p.) attenuated the loss of MNCV in a dose-dependent manner in the treatment groups by 63.78 ± 2.35*m/s and 86.70 ± 4.32*m/s, respectively.

## Discussion

In the present study, catechin was evaluated as an alternative treatment option for neuropathic pain. Therefore, we conducted this study using animal models to further validate and evaluate its practicality in neuropathic pain. The antinociceptive effects of the drug were tested using a CCI model. Due to the similarity of rat behavioral responses to human pathophysiology and the pursuit of effective therapies, the CCI-induced neuropathy model was used to carry out the experiment. It is used as a target for the discovery of new therapeutics by different researchers (Le Coz et al., 2017; Limcharoen et al., 2020; Fonseca-Rodrigues et al., 2021).

The present study has shown a decrease in the activation of NF-κβ extracted from the sciatic nerve in a dose-dependent manner at given catechin doses. This outcome is consistent with an earlier study by Zulazmi et al. (2015). Catechin at given doses has shown a significant decrease in the expression of these pro-inflammatory cytokines. The improvement in allodynia and spontaneous pain in the last 2 weeks can be attributed to this corresponding decrease in the expression of TNF-α, IL-6, and IL-β.

Catechin reversed the signs of neuropathic pain, although only in the late phase of neuropathic pain. Catechins were able to significantly reduce hyperalgesia and allodynia in a dose-dependent manner after treatment. Treatment with catechins strongly modulates the expression of cytokines in the sciatic nerve. The increased TNF-α, IL-6, and IL-β levels were completely restored to normal levels in the rat brain by catechin treatment. Again, we observed a correlation between the effect on pain and cytokines. The results suggest that catechin can be considered a parent compound that blocks NF-κβ activation. It showed that the attenuation of pain symptoms and decreased levels of NF-κβ–regulated inflammatory cytokines (TNF-α, IL-6, and IL-β) in the chronic constriction injury model. Similar results have been observed by Gopalsamy et al. (2017).

These data indicate that CCI causes inflammation in neurodegenerative pain through various mechanisms. Modulation of inflammatory molecules appears to be a common feature achieved through different mechanisms, and the NF-κβ pathway has been suggested to play an important role in neuropathic pain. It should be noted that the results of the current study show consistency between all parameters, and catechin can be considered an effective treatment for neuropathic pain.

## Conclusion

Neuropathic pain is a serious disorder that has a significant impact on the quality of life of people who suffer from it, despite the fact that treatment options are limited. The treatment of neuropathic pain remains a major clinical challenge due to the complex mechanisms involved in the development of neuropathic pain.

In conclusion, we have demonstrated that the oral administration of catechin reduced CCI-induced neuropathic pain symptoms in rats. Based on the evidence-based result obtained in this research, it is possible to draw meaningful conclusions with catechin as an alternative therapy for neuropathic pain. Our data suggest that catechin blocks NF-κβ activation in the rat model with neuropathic pain. It further decreases the levels of NF-κβ-regulated inflammatory cytokines (TNF-α, IL-6, and IL-β).

The analgesic effect of catechin-mediated activation of NF-κβ in a rat model of neuropathic pain requires further research in different animal models to understand the exact mechanism of anti–TNF-α therapies. Validation of the results will offer hope for future success in treating neuropathic pain with maximum pharmacological effects and fewer side effects. Oral administration of catechin might represent a therapeutic perspective in managing neuropathic pain conditions.

## Data Availability

The raw data supporting the conclusions of this article will be made available by the authors, without undue reservation.
